# Exploring the potential synergistic pharmacological and psychological effects of caffeine on exercise performance: a placebo-balanced study

**DOI:** 10.5114/biolsport.2026.153332

**Published:** 2025-08-05

**Authors:** Juan Del Coso, Beatriz Lara, César Gallo-Salazar, Francisco Areces, Millán Aguilar-Navarro, Verónica Giráldez-Costas, Jorge Gutiérrez-Hellín, Fernando Valero, Juan Jose Salinero

**Affiliations:** 1 Sport Sciences Research Centre, Rey Juan Carlos University, Fuenlabrada, Spain; 2 Exercise Physiology Laboratory (GIDECS) Faculty of Health Sciences – HM Hospitals, University Camilo José Cela, Villanueva de la Cañada, España; 3 Instituto de Investigación Sanitaria HM Hospitales, Madrid, Spain; 4 Faculty of Health Science, Universidad Francisco de Vitoria, Madrid, Spain; 5 Sport Training Laboratory (GIRD), Faculty of Sport Sciences, University of Castilla-La Mancha, Toledo, Spain

**Keywords:** Placebo effect, Deception, Ergogenic aids, Nutrition, Sport supplements

## Abstract

Research specifically investigating the distinct pharmacological and expectancy effects of caffeine using the deceptive placebo-balanced design remains scarce and with contradictory results. The aim of this study was to investigate the potential synergy between the pharmacological and expectancy effects of caffeine on exercise performance by using a placebo-balanced design. Sixteen physically trained athletes (11 males and 5 females; 21.7 ± 5.0 yr) participated in a study with a deceptive protocol including four randomized conditions: placebo informed–placebo ingested (control); placebo informed–caffeine ingested (pharmacological effect); caffeine informed–placebo ingested (expectancy effect); and caffeine informed–caffeine ingested (combined effects). Sixty minutes after ingestion, participants performed a countermovement jump, a standing triple jump, a medicine ball throw and a 20-m running sprint test. Relative to control, the pharmacological effect trial increased standing triple jump distance (+2.1% p = 0.032; Cohen’s *d*= 0.59) and reduced 20-m sprint time (-0.8% p = 0.030; Cohen’s *d*= 0.60). The combined effect trial reduced 20-m sprint time (-0.8% p = 0.021; Cohen’s *d*= 0.64) in comparison to control. The expectancy effect trial did not modify performance in any of the performance tests with respect to control. When averaging the performance across all four tests, improvements relative to the control trial were +0.9%, +0.6% and +1.3% for pharmacological, expectancy and combined effects, respectively. The ingestion of caffeine, whether or not participants expected to receive it, improved exercise performance. This suggests that the primary driver of caffeine’s ergogenic effect is its pharmacological action, with only a minor contribution from expectancy.

## INTRODUCTION

The placebo effect is a desirable outcome resulting from a person’s belief that they have ingested a potentially beneficial substance, which subsequently promotes a positive response [1]. The placebo effect can occur even without ingesting the substance, or it may amplify the benefits when the substance is actually consumed. The placebo effect has been extensively studied for decades in medicine, psychology, and neuroscience and is widely recognized as a powerful neurobiological phenomenon that can amplify the perceived effects of various substances [2]. In sports sciences, the placebo effect has been less thoroughly investigated, despite its potential to benefit athletes using supplements and performance-enhancing substances. While various substances may yield exercise performance benefits through the expectation of their effects [3], caffeine is the substance most commonly associated with the placebo effect, due to its widely recognized performance-enhancing properties across various sports disciplines [4–6].

The ergogenic effects of caffeine have been well-documented in numerous double-blind, placebo-controlled studies [7, 8], and through meta-analyses of the main performance outcomes of these investigations [5, 9]. In these studies, the experimental protocol typically involves two identical trials: one following caffeine ingestion and the other following the ingestion of a placebo, with exercise performance measurements approximately one hour after substances intake [4]. In most of these experiments, the order of caffeine and placebo is randomized, and both participants and researchers remain blinded to the substance tested on each day, ensuring a double-blind control design. This type of experiment is widely used in sports science research on caffeine to control for its potential placebo effect. Therefore, these double-blind, placebocontrolled experiments are used to isolate the pharmacological effect of caffeine on exercise performance [10]. The findings of these investigations underscore the strong ergogenic effects of caffeine that occur independently of psychological expectancy, although the placebo effect may still contribute to performance enhancement. This is because both caffeine and the belief in having consumed caffeine may share dopaminergic mechanisms of action, and expectation related to caffeine intake can modulate both psychological and physiological responses [11]. Psychologically, the expectation of having consumed caffeine may influence mood, alertness, and perceived exertion. Studies have demonstrated that participants who believe they have ingested caffeine report increased vigour and attention, even when only receiving a placebo [12]. From a physiological perspective, the expectation of caffeine consumption may induce several changes in autonomic responses, such as dopaminergic responses [11], or reduced resting heart rate [13]. However, these physiological responses do not necessarily translate into significant performance enhancements. Additionally, despite the methodological quality of this type of investigation, they may not reflect the true potential benefits of caffeine supplementation in real-world sports contexts [4]. This is because athletes may experience a combined pharmacological-psychological effect of caffeine as the potential benefits of substance intake may be increased by the expectancy of caffeine’s ergogenicity [14].

Investigating the pharmacological and psychological effects of caffeine (both, in isolation and in combination) requires a deceptive experimental design, in which participants are intentionally misinformed about whether they have ingested caffeine or a placebo. This approach is essential to disentangle the pharmacological effects from expectancy-driven responses. The appropriate experimental design for this purpose is called the “placebo-balanced” design [15]. It involves four trials to control for both expectancy and actual caffeine intake: (1) participants receive an informed placebo (control condition); (2) participants receive caffeine but are misinformed, believing it is a placebo (isolating the pharmacological effect); (3) participants receive a placebo but are told it is caffeine (isolating the expectancy or placebo effect); and (4) participants receive caffeine and are correctly informed, to assess the potential synergy of pharmacological and psychological effects. Research specifically examining the distinct pharmacological and psychological effects of caffeine on exercise performance using the placebo-balanced design remains limited and contradictory [14, 16]. Foad et al. [16] concluded that the ergogenic effects of caffeine during a 40 km time trial were primarily driven by its pharmacological properties (i.e., when the substance was ingested regardless of whether the participant believed that caffeine had been ingested the substance) with no additive expectancy/placebo effect. On the contrary, Hurst et al. [14] found that believing to have ingested caffeine improved performance to the same magnitude as actually receiving caffeine, suggesting that most of the ergogenic effect of caffeine is psychological.

In the literature, there are other studies with simpler experimental designs using just a deception for inducing the placebo effect of caffeine (i.e., informing participants about caffeine ingestion when in fact only ingested a placebo) [13, 17, 18]. These investigations have reported several physiological and performance benefits associated with caffeine expectancy [13, 17] although not in all cases [18]. However, these latter studies do not allow to differentiate the contribution between the pharmacological effect of caffeine and its potential psychological benefits derived from expectancy and in most cases, no actual ingestion of the substance was produced.

While it is generally acknowledged that caffeine has a placebo effect on exercise performance derived from expectancy [4], the extent to which this effect contributes to its overall ergogenic benefits, when both ingestion and expectancy are combined, remains unclear. To address the gap in the literature, this study aimed to explore the potential synergy between the pharmacological and expectancy effects of caffeine on exercise performance, utilizing a balanced placebo design with a deceptive element.

## MATERIALS AND METHODS

### Design

**FIG. 1:**
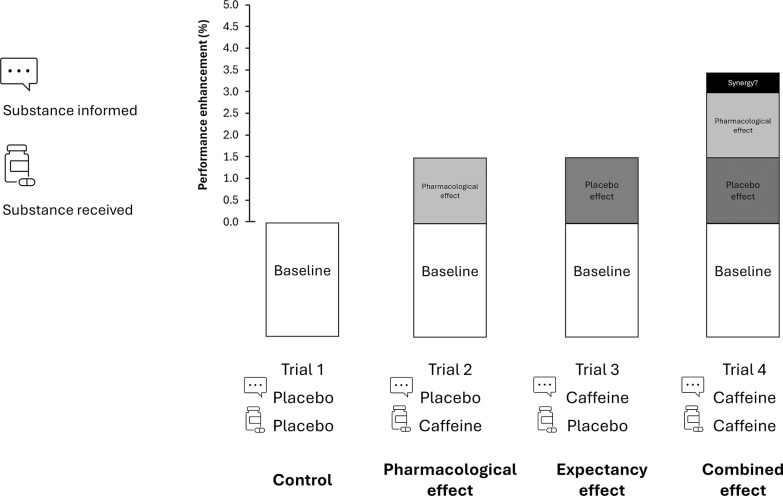
Theoretical approach to the ergogenic effect of caffeine in sport. When caffeine is ingested in a real sporting context, the performance benefits may be derived from the pharmacological effects of the substance (such as the blockade of adenosine receptors) added to the psychological effects derived from the expectancy of ingesting an ergogenic aid (such as anticipated increased performance or reduced fatigue). Although in the real sporting context both potential effects are obtained together, the use of a placebo-balanced study, with a deceptive protocol, may help to isolate the pharmacological and expectancy effects to assess the total performance benefit derived from caffeine intake. Through the combination of ingestion of an active (caffeine) and a non-active (placebo) substances and after receiving true or deceived information about the substance ingested, the pharmacological effect (i.e., participant receives caffeine but is informed that has ingested a placebo), the placebo effect (i.e., the participant receives a placebo but is informed that has ingested caffeine) and the combined effect of caffeine intake (i.e., the participant receives caffeine and is informed that has received caffeine) can be obtained by comparison with a baseline situation (i.e., the participant receives placebo and is informed that has received a placebo).

A repeated, randomized, and counterbalanced experimental design was used to compare the pharmacological and psychological effects of caffeine in isolation or in combination on exercise performance. Four conditions were established to analyse the effect of deceptive or actual caffeine and placebo ingestion and their interaction: 1) placebo informed – placebo ingested, established as the control condition; 2) placebo informed – caffeine ingested, to isolate the pharmacological effect caffeine (pharmacological); 3) caffeine informed – placebo ingested to test the psychological effect of caffeine derived only from expectancy (expectancy); 4) caffeine informed – caffeine ingested to combine both effects (combined). The four trials were performed under the same conditions and only the substance actually ingested, and the information of the substance ingested varied among conditions. In trials with caffeine intake, an opaque and unidentifiable capsule filled with a dose of 3 mg per kg of body mass of caffeine (3 mg/kg; HSN, Spain) was provided. We selected 3 mg/kg of caffeine because this dose has been proven effective in inducing performance benefits on jumping, sprints and throwing activities [5, 7, 19]. On the days with the placebo, the same capsule was ingested but filled with a placebo substance (cellulose; Guinama, Spain). Figure 1 displays the study design.

### Participants

Sixteen physically trained adults volunteered to participate in this experimental study (11 males and 5 females; 21.7 ± 5.0 years old; 71.4 ± 8.3 kg of body mass; 175.9 ± 7.8 cm). They all had no physical limitations or musculoskeletal injuries that could affect exercise performance at the moment of the experiment. All of the participants fulfilled the following inclusion criteria: a) age between 18 and 40 years old; b) caffeine naïve, low or mild habitual caffeine consumption (< 2.99 mg/kg/day); and c) regular physical training > 2 days per week in power-based sport and exercise disciplines as team sports or resistance training. Participants were excluded if they reported a) injury within the previous three months; b) a positive smoking status; c) medication usage within the previous month; d) a previous history of cardiopulmonary diseases; e) allergy to caffeine; or f) use of oral contraceptive pills. They were encouraged to maintain a balanced diet throughout the duration of the experiment following previous nutritional guidelines to assure carbohydrate and protein availability and correct hydration during the whole experiment [20–22]. Participants were also instructed to continue their training routines throughout the duration of the study to avoid any detraining effects and to refrain from vigorous exercise for at least 48 hours prior to each experimental trial. Last, all subjects were asked to abstain from any form of dietary caffeine intake and from dietary supplement use for the duration of the study. An *a priori* sample size calculation estimated a minimum of 10 individuals based on the effect size of caffeine equal to 1.19 Cohen’s units based on a previous study that reported improvements in jump performance of such magnitude with 3 mg/kg body weight of caffeine [19]. The G*Power software (v.3.1.9., Germany) was used for sample size calculation, considering a repeated measures design aiming for a statistical power of 95% and an α error of 0.05. In case of drop out, and to allow a counterbalanced order of the trials, sixteen participants were initially recruited, and all of them completed the experiment. Before the study, all participants were informed about the testing protocols and possible risks involved and were invited to provide written informed consent. The study was performed following the principles of the Declaration of Helsinki, and the experimental protocols were approved by the local ethics committee (ref. 28.1.2021CEIUCJC). Habitual caffeine intake was measured by using a modified version of the validated questionnaire by Bühler et al. [23], and participants’ categorization was obtained using the classification suggested by Filip et al. [24].

### Procedures

All participants participated in all four experimental conditions and acted as their own controls. Additionally, participants were familiarised with the testing procedures employed in the current experiment through a familiarisation session performed one week before the first trial. In this same familiarization session, anthropometric data were obtained to calculate caffeine dosage and habitual caffeine intake was obtained through a questionnaire [23]. All performance measurements were performed at the same time of day to avoid the effects of circadian rhythms in an indoor sports facility, with similar temperature and humidity conditions (≈19ºC, 58%, respectively). The recovery between trials was set to 3–5 days to allow recovery and caffeine wash-out [4]. All participants performed the four testing conditions within a 15-day period.

Before the study onset, participants were informed about the ergogenic properties of caffeine including its effect on a wide variety of sports situations, including sprints, jumps and throwing events. To produce a deceptive element, participants were told they would participate in 4 trials, two with caffeine intake and two with placebo intake with the aim of measuring the intra-subject reliability of caffeine’s ergogenicity. This information was true with the exception that one day with each substance the information about the substance ingested was misleading to disentangle the pharmacological and expectancy effects of caffeine. Only one researcher was aware of the true substance tested in each day (and he did not participate in any performance measurement) while the other researchers were blind to the substance under investigation on each day.

On the day of each experimental trial, the participants arrived at the facility between 12.30 and 2.30 pm in a fed state (≈3 hours after their last meal, which was the same before all trials). Upon arrival, the capsule with the experimental treatment (caffeine or placebo) was provided and ingested with 250 mL of water. At this moment, the researcher informed the participant about the substance under investigation that day, following the procedures described above. Forty-five minutes after this, participants performed a 15-min standardized warm-up. The warm-up consisted of 2 × 20 m repetitions of running drills (including ‘A’ skips, ‘B’ skips, straight-leg runs, butt kicks, and alternate hop-and-step jumps), 2 × 30 m sub-maximal sprints, 3 sub-maximal countermovement jumps and 3 × 5 throws against a wall and 5 submaximal frontal throws. Just 60 min after substance ingestion, the participants performed the following performance tests with a recovery time of 5 min between tests and always in the same order. A standardized verbal encouragement was used in performance tests to aid in obtaining maximum performance in each condition.

### Countermovement jump

Participants were positioned on a wireless dual force plate system with a sample rate of 1000 Hz (Hawkin Dynamics Inc.) with both feet at shoulders width, body weight evenly distributed and hands on the waist. On the command, they performed the jump by first descending into a squat position and performing a rapid extension of the knees and hips to propel themselves upward to reach maximal height. Jump height was obtained from the change in the system centre of mass position between the instant of take-off and peak positive vertical displacement of the system centre of mass, calculated using the vertical velocity of the system centre of mass at the instant of take-off and the equations of uniformly accelerated motion. Participants performed 3 repetitions of this jump (with 1 min of recovery between repetitions), and data of the jump with the highest height was used for statistical analysis.

### Standing triple jump

Participants started from a stationary position behind a line and with their feet shoulder-width apart. On command, the participants initiated the test by performing the first jump using the propulsion of their dominant leg while the non-dominant leg was flexed and displaced forward for ground contact. Participants quickly transitioned from the first jump to the second and third jumps by alternatively absorbing the landing force with flexed knees and immediately pushing off the ground to reach maximal distance in each jump while maintaining balance and rhythm. The last jump was finished with a landing with both feet at the same time. Participants freely swung their arms backward and forward for momentum and for balance. The total distance of the jump was measured from the starting line to the furthest point of landing of the last jump using a metric tape. Participants performed 3 repetitions of this jump (with 1 min of recovery between repetitions) and data of the jump with the longest distance was used for statistical analysis.

### Medicine ball throw

Participants started from a stationary position behind a line and with their feet shoulder-width apart. and holding the medicine ball over the head (3 kg for men and 2 kg for women). From this position, the participants engaged explosively the knees, hips, core, shoulders and arms to create the maximum torque for releasing the ball over the head in a trajectory for maximal distance. The distance from the release line to the furthest point of ball landing was obtained through a metric tape. Participants performed 3 repetitions of this throw (with 1 min of recovery between repetitions) and data of the throw with the longest distance was used for statistical analysis.

### 20-m running sprint

Participants started from a stationary position behind a line and with the foot of the dominant leg behind the non-dominant leg for propulsion. On participants ran at maximal speed for 20 m in a straight line, and the time needed to cover the distance was measured using 2 photocell gates (dual-beam, with the trigger criterion being the first occurrence of both beams being broken, Witty-Gate, Microgate, Italy) placed 1 m and 1.20 m above the ground [25]. Each sprint was initiated with participants 1 m behind the first photocell gate and the digital timer was automatically initiated when the participant crossed the first gate, and it was automatically stopped when crossing the second gate. The best performance out of 2 repetitions was recorded for subsequent analysis, and a 2-minute resting period was allowed between repetitions.

All tests demonstrated good test-retest reliability, with ICC > 0.90 and CV < 5% [26]. After completing the performance tests, participants resumed their daily activities while being reminded to avoid caffeine-containing foods and beverages. The morning after each experimental trial, participants received a weblink on their personal smartphones to fill out an electronic form on possible side effects. In this questionnaire, participants had to indicate the magnitude of side effects typically associated to caffeine intake such as nervousness, increased diuresis, digestive problems, or insomnia with a 1- to 10-point scale. Participants were previously informed that 1 point meant the minimal magnitude of the side effect and 10 points meant the maximal magnitude of the side effect. This questionnaire was previously used to assess the side effects derived from caffeine ingestion in the sport domain [27].

### Data analysis

**FIG. 2:**
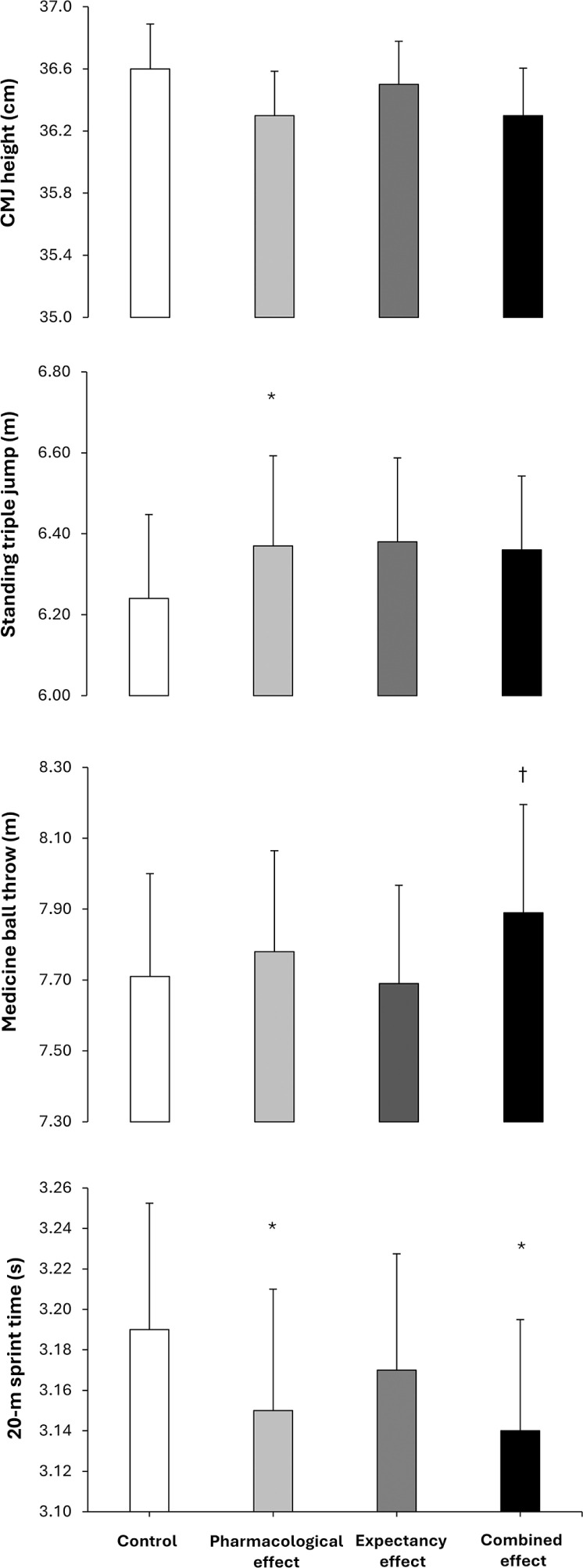
Jump height in a countermovement jump (CMJ), distances reached in a standing triple jump and a medicine ball throw and time to complete a 20-m sprint test in a placebo-balanced study with a deceptive protocol including four randomized conditions: 1) placebo informed–placebo ingested (control condition); 2) placebo informed–caffeine ingested (pharmacological effect); 3) caffeine informed–placebo ingested (expectancy effect); and 4) caffeine informed–caffeine ingested (combined effect). * depicts a statistical difference with respect to control, at p < 0.050. † depicts a statistical difference with respect to expectancy condition, at p < 0.050.

**FIG. 3:**
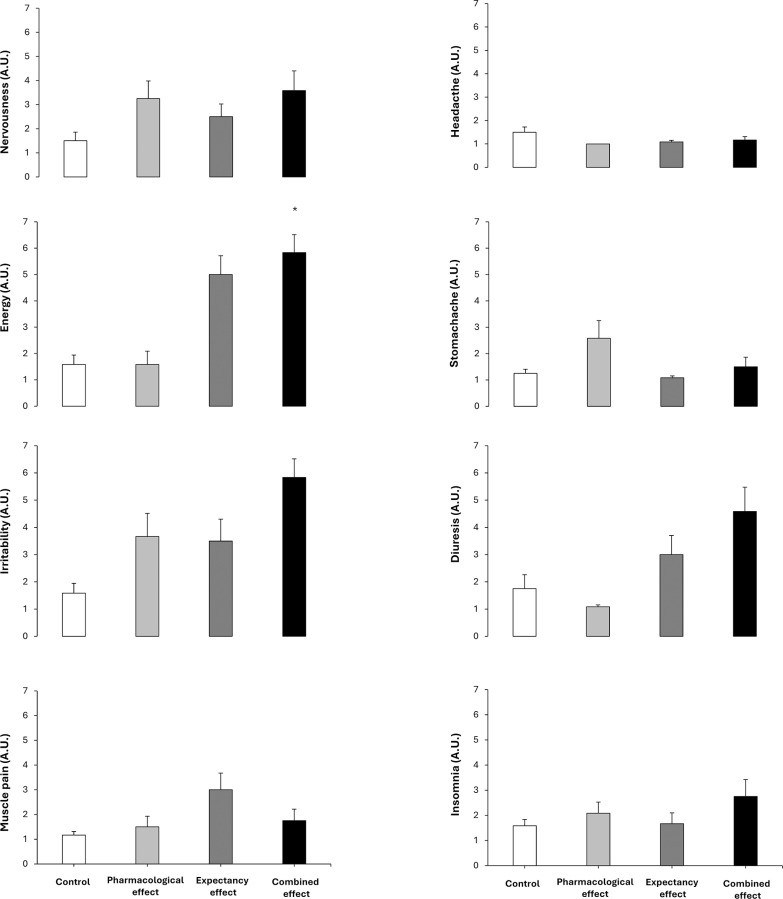
Side effects in a placebo-balanced study with a deceptive protocol including four randomized conditions: 1) placebo informed– placebo ingested (control condition); 2) placebo informed–caffeine ingested (pharmacological effect); 3) caffeine informed–placebo ingested (expectancy effect); and 4) caffeine informed–caffeine ingested (combined effect). * depicts a statistical difference with respect to control, at p < 0.050.

Data are presented as mean ± standard deviation for each condition. Initially, the Shapiro-Wilk test was used to confirm the normality of the raw data. For exercise performance variables, a two-way factorial ANOVA with repeated measures (2 × 2; substance received = caffeine *vs.* placebo received × substance informed = caffeine *vs.* placebo informed) was conducted to identify the main effects of substance received, substance informed, and their interaction (data presented in Table 1). After this, one-way ANOVA with Least Significant Difference (LSD) adjustment was conducted for pairwise comparisons and 95% confidence intervals for differences were calculated (data presented in Figure 2). Cohen’s *d* effect size (ES) was also calculated for the comparison of control with all the other treatments in the exercise performance variables and were interpreted as trivial (< 0.2), small (< 0.6), moderate (< 1.2), large (< 2) and very large (> 2) [28]. Data was analysed using SPSS (v29.0). Performance enhancement (in percentage) with respect to control was calculated for all the remaining treatments ([treatment-control]/control) for each test and as an average of the performance benefits obtained in the four tests employed in this investigation. The nonparametric Friedman test was performed to analyze the difference between conditions for side-effects variables, with Wilcoxon pairwise comparisons (data presented in Figure 3). Wilcoxon r effect size was also calculated for the pairwise comparisons and were interpreted as small (< 0.1), medium (< 0.3) and large (< 0.5). In all statistical tests, a level of p < 0.050 was set to establish statistically significant differences.

**TABLE 1:** Results of the factorial ANOVA of the main effects of substance received and substance informed on exercise performance tests.

Main effect	Substance received^*^		Substance informed^**^	
Variable (units)	Placebo	Caffeine	Placebo	Caffeine
CMJ height (cm)	36.5 ± 7.0	36.3 ± 8.0	36.4 ± 7.7	36.4 ± 7.3
Standing triple jump (m)	6.31 ± 0.82	6.36 ± 0.80	6.30 ± 0.85	6.37 ± 0.77
Medicine ball throw (m)	7.70 ± 1.12	7.84 ± 1.16	7.74 ± 1.13	7.79 ± 1.15
20-m sprint time (s)	3.18 ± 0.24	3.15 ± 0.23	3.17 ± 0.24	3.15 ± 0.23

* For the main effect of the substance received, placebo reflects data from the two trials with placebo ingestion (control condition and expectancy condition) and caffeine reflects data from the two trials with caffeine intake (pharmacological condition and combined condition), irrespective of the information given to the participants.

** For the main effect of substance informed, placebo reflects data from the two trials where participants were informed that they had received a placebo (control condition and pharmacological condition) and caffeine reflects data from the two trials where participants were informed that they had received caffeine (expectancy condition and combined condition), irrespective of the substance actually ingested.

## RESULTS

Table 1 contains data from the factorial ANOVA employed to calculate the main effects of substance received and substance informed. In the CMJ test, there was no main effect of substance received (F = 0.163; p = 0.692), substance informed (F = 0.0001; p = 0.992) or interaction between these effects (F = 0.013; p = 0.911) on the height reached. Jump height was similar irrespective of the substance ingested or the substance informed and there were no differences in the pairwise comparison between the four experimental conditions (Figure 2).

In the standing triple jump, the main effect of substance received was close to statistical significance (F = 3.305; p = 0.089) with no main effect of substance informed (F = 0.965; p = 0.341) or interaction between these effects (F = 2.859; p = 0.112) on the distance reached during the jump. The main effects on jump distance tended to be higher with caffeine ingestion (+0.8%) in comparison to placebo ingestion. In the pairwise comparisons, the pharmacological effect trial increased standing triple jump distance (+2.1%; CIdiff [0.01–0.25 m]; p = 0.032; Cohen’s *d* = 0.59) over control with no other difference between trials (Figure 2).

In the medicine ball throw, there was a tendency for a main effect of substance received (F = 4.030; p = 0.063) with no main effect of substance informed (F = 0.539; p = 0.474) or interaction between these effects (F = 1.429; p = 0.250) on the distance reached during the throw. The main effects on throwing distance tended to be higher with caffeine ingestion (+1.8%; Table 1). In the pairwise comparison, the combined effect trial increased throw distance (+2.5%; CIdiff [0.00–0.39 m]; p = 0.050; Cohen’s *d* = 0.53) over the expectancy trial with no other difference between trials.

In the 20-m sprint test, there was a main effect of substance received (F = 6.610; p = 0.021) with no main effect of substance informed (F = 1.614; p = 0.223) or interaction between these effects (F = 0.455; p = 0.510) on time employed to cover the distance. The main effect of substance indicated that sprint time was shorter with caffeine ingestion (-0.9%; Table 1). In the pairwise comparison the pharmacological effect trial (-0.8%; CIdiff [0.004–0.076 s]; p = 0.030; Cohen’s *d* = 0.60) and the combined effect trial reduced sprint time (-0.8%; CIdiff [0.008–0.087 s]; p = 0.021; Cohen’s *d* = 0.64) over the control trial.

There were no main effects of substance received, substance informed or interaction between these effects for nervousness, irritability, muscle pain, headache, stomachache, diuresis, or insomnia (all p > 0.050; Figure 3). Only a main effect of substance informed was found for energy (p = 0.003). In the pairwise comparison, only the combined effect trial increased the energy over the control trial (p = 0.009; Wilcoxon *r* = 1.1 large).

## DISCUSSION

The aim of this study was to investigate the potential synergy between the pharmacological and expectancy effects of caffeine on exercise performance by using a placebo-balanced design. The main outcomes of this study reveal that the pharmacological effect of caffeine is the main contributor of the ergogenicity of the substance on short-term all-out exercise performance with a minor contribution of caffeine expectancy. This is because the pharmacological effect increased the distance reached during a triple jump and reduced 20-m sprint time, while the combined effect (pharmacological + expectancy) reduced 20-m sprint time in comparison to the control condition. However, the expectancy effect did not modify performance in any of the tests. No significant differences were found in side effects, except for an increase in the magnitude of the feeling of energy in the combined effect condition compared to the control condition. These findings suggest that, in the context of short-term high-intensity exercise, caffeine’s ergogenic effects are primarily pharmacological, with minimal contribution from expectancy.

Some studies with deceptive designs to induce caffeine expectancy (i.e., the placebo effect of caffeine) have yielded some performance benefits associated to the belief of caffeine ingestion [13, 17, 29–32]. These studies demonstrated that the placebo effect of caffeine enhances exercise performance, even when caffeine is not actually ingested. However, these studies have been misinterpreted to suggest that the performance benefits of caffeine supplementation arise solely from psychological advantages, rather than from the physiological effects of actual caffeine consumption, or from a combination of both factors. This is because, to investigate pharmacological and psychological effects of caffeine separately, it is needed to employ a placebo-balanced experimental design, a complex type of experiment with four conditions used only in two experiments to the date [14, 16]. The scarcity of studies using the placebo-balanced design to assess caffeine’s ergogenicity is compounded by another limitation: these studies measured exercise performance with only one test. Given the certain intra- and inter-individual variability in the response to caffeine intake [33, 34], it is probable that the use of only one performance test has contributed to the disparity in the results regarding the contribution of the expectancy/pharmacological effects of caffeine on isolation and in combination [16].

The current study showed that the trial with placebo informedcaffeine ingested (pharmacological effect) increased the distance reached during a triple jump and reduced 20-m sprint time while the trial with caffeine informed-caffeine ingested (combined effect) reduced 20-m sprint time in comparison to the control condition (i.e., placebo informed-placebo ingested). Interestingly, these two trials (pharmacological and combined) shared the actual ingestion of 3 mg/kg of caffeine, one with deceiving information about the substance ingested and the other with current information about the substance ingested. Additionally, in all performance tests, the main effect of caffeine ingestion was always superior to the main effect of caffeine expectancy (Table 1). These data suggest that caffeine ingestion—regardless of whether individuals expect to receive it—enhances exercise performance. This coincides with all the evidence gained with dozens of placebo-controlled and double-blinded studies that found performance benefits of caffeine even when controlling for the placebo effect of the substance [5, 9]. Of note, the trial with caffeine informed-placebo ingested (expectancy), which was used to induce the placebo effect of caffeine, did not modify performance in any of the performance tests suggesting the limited efficacy of caffeine expectancy in this study. Collectively, the performance benefits measured in this investigation point toward the pharmacological effect of caffeine as the main contributor of the ergogenicity of the substance on exercise performance with a minor contribution of caffeine expectancy.

**FIG. 4:**
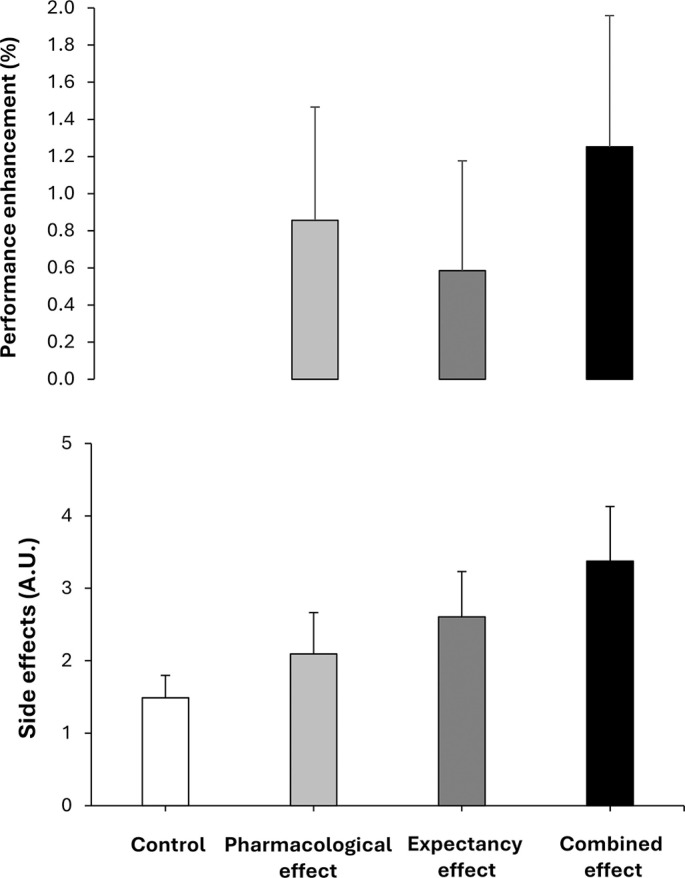
Overall performance benefits and side effects in a placebobalanced study with a deceptive protocol including four randomized conditions: 1) placebo informed–placebo ingested (control condition); 2) placebo informed–caffeine ingested (pharmacological effect); 3) caffeine informed–placebo ingested (expectancy effect); and 4) caffeine informed–caffeine ingested (combined effect). The performance benefit represents the average of the changes produced by each condition over control (baseline) when analysing the data of 4 performance tests (countermovement jump, standing triple jump, medicine ball throw and 20-m sprint test). The side effect represents the average of the changes produced by each condition over control (baseline) when analysing the data of 8 side effects (nervousness, energy, irritability, muscle pain, headache, stomachache, diuresis and insomnia).

Figure 4 contains the mean performance benefit obtained in each condition, with respect to the control condition. This figure depicts the potential addition/synergy between the pharmacological and expectancy effects of caffeine for both performance and side effects. In terms of magnitude, the performance benefit when testing the pharmacological effect of caffeine (+0.9%) was superior to when testing the placebo effect in isolation (expectancy, +0.6%). When both effects were tested together in the combined effects trial, the magnitude of the performance benefit further increased (+1.3%); however, it did not reach the level of the combined effects when assessed individually. This suggests that both the pharmacological and expectancy effects of caffeine worked together to produce a greater benefit; however, we cannot classify this as an additive or synergistic effect. From a practical perspective, those sports scientists and practitioners seeking for performance benefits from caffeine supplementation in their athletes should combine the use of caffeine with awareness transmitted to their athletes about the potential expected benefits. This seems the best approach to maximizing the ergogenic benefits of caffeine on exercise performance. On the contrary, the induction on the placebo effect of caffeine seems less effective than the actual ingestion of the substance to obtain performance benefits.

With respect to side effects, only the combined effect trial produced an increase in the magnitude of the feeling of energy compared to the control trial with no significant differences observed in the remaining variables (Figure 3). Although increased energy may not be considered an adverse effect, elevated energy levels following exercise could be relevant in contexts where caffeine is consumed to enhance performance, as they may influence recovery or negatively affect sleep, particularly when ingestion occurs in the late afternoon or evening [27]. Additionally, the magnitude of the side effects experienced by the participants reported in Figure 4 confirms a comparable pattern to the one found for performance. In terms of magnitude, the intensity of the side effects when testing the pharmacological effect of caffeine (+28%) was superior to when testing the placebo effect in isolation (expectancy, +17%) and both lower to these effects tested in combination (i.e., combined, +83%). These data reveal that the pharmacological side effects of caffeine ingestion or the potential negativity of the placebo effect of caffeine are minor than the drawbacks experienced by athletes when they ingest caffeine in real sports contexts. In this case, the potential negative effects of the substance intake and the expectancy of side effects (as athletes are habitually aware that caffeine produce some side effects as nervousness, anxiety and insomnia) produce a synergistic consequence to almost duplicate the intensity of the side effects.

### Limitations and future directions

Despite the complexity of the current investigation, it still possesses several limitations. First, we tested only one dose of caffeine (i.e., 3 mg/kg) to activate both pharmacological and expectancy effects of caffeine. Although it is unlikely that a higher dose would have amplified the pharmacological effect in terms of performance [35], future research should explore whether the mere knowledge of ingesting a higher dose could elicit a greater expectancy effect [32]. Second, the sample was composed of physically trained individuals with low habituation to caffeine as they have low or mild daily intake of caffeine. So, the outcomes of this study may not be transferable to athletes habituated to caffeine as they may experience both lower performance benefits from acute caffeine supplementation [36] but also side effects of lower magnitude [37]. In addition, participants’ previous experience with caffeine may have influenced the degree to which they believed or doubted the information provided in each condition. However, we were unable to verify their beliefs directly. Due to the use of a balanced placebo design, in which participants were intentionally misinformed about the content of the supplement in two of the four conditions, asking them to report what they believed they had consumed could have revealed the deception and compromised the integrity of the blinding. Third, although we included four differential tests to enhance the assessment of the potential performance benefits derived from the pharmacological and expectancy effects of caffeine, all these tests were short-term all-out performance measurements that measured predominantly muscle power. So, further investigations are needed to establish the isolated and combined pharmacological and expectancy effects of caffeine supplementation in longer trials with a more evident aerobic component. Fourth, due to the characteristics of the experimental design of this study (comprising four trials with 3 to 5 days of recovery between each, spanning a total duration of 15 days), it was not feasible to schedule the experimental sessions in a way that ensured all female participants were in the same phase of their menstrual cycle during each measurement. However, previous research has reported similar ergogenic effects of caffeine on anaerobic performance in both male and female athletes [8]. Furthermore, no significant differences have been observed in the ergogenic effects of caffeine based on menstrual cycle phase [38–40]. Taken together, this evidence suggests that the potential performance benefits of acute caffeine intake may be equally present in female athletes, regardless of their menstrual cycle phase. Last, no genetic testing was employed in our participants to determine variants that may potentially affect responses to caffeine intake such as the -163C > A (rs762551) in the *CYP1A2* gene or the 1976C > T (rs5751876) in the *ADORA2A* gene.

Finally, while the quantitative results of this study provide key insights into the pharmacological and expectancy effects of caffeine on exercise performance, incorporating qualitative assessments could further enhance understanding of individual variability in responses to caffeine and placebo conditions. Collecting subjective reports on factors such as participants’ perceived physical capabilities, pre-exercise readiness, and motivation across conditions could help clarify the contribution of psychological mechanisms to performance outcomes.

### Practical applications

Exercise practitioners incorporating caffeine supplementation into their athletes’ routines may benefit from not only optimizing the dose, timing, and source of caffeine, but also from informing athletes about its potential performance-enhancing effects. The results of this study advise against the use of the placebo effect of caffeine (without actual ingestion of the substance) as the magnitude of the performance benefits is always inferior to the intake of the substance. In addition, understanding the potential negativity of caffeine supplementation is crucial to decide whether the substance should be recommended in each sporting context. The benefit (increases in performance between 1–4%) should be contextualized in the risk of suffering mild side effects such as excessive energy post-exercise.

## CONCLUSIONS

The ingestion of caffeine, whether or not participants expected to receive it, improved several exercise performance tests while the induction of the placebo effect did not produce any performance advantage. Overall, the magnitude of the benefits of caffeine ingestion was superior over the benefits of caffeine expectancy, suggesting that the primary driver of caffeine’s ergogenic effect is its pharmacological action, with only a minor contribution from expectancy. Still, the ergogenic effect of caffeine is a composite of the pharmacological action of the drug and the psychological benefits obtained from the knowledge that caffeine is ergogenic, without an additive or synergistic actions. Last, for most caffeine-associated side effects, the addition of the pharmacological action of caffeine and the expectancy of side effects from this stimulant produced an effect of higher magnitude that these same effects isolated.

## References

[1] Colloca L, Barsky AJ. Placebo and Nocebo Effects. N Engl J Med. 2020; 382:554–61. doi: 10.1056/NEJMra1907805. 32023375

[2] Beedie C, Hettinga F. Introduction to the special edition on the placebo effect in sport and exercise. Eur J Sport Sci. 2020; 20(3):277–8. doi: 10.1080/17461391.2020.1757682. 32299310

[3] Beedie C, Benedetti F, Barbiani D, Camerone E, Cohen E, Coleman D, et al. Consensus statement on placebo effects in sports and exercise: The need for conceptual clarity, methodological rigour, and the elucidation of neurobiological mechanisms. Eur J Sport Sci. 2018; 18(10):1383–9. doi: 10.1080/17461391.2018.1496144. 30114971

[4] Guest NS, VanDusseldorp TA, Nelson MT, Grgic J, Schoenfeld BJ, Jenkins NDM, et al. International society of sports nutrition position stand: caffeine and exercise performance. J Int Soc Sports Nutr. 2021; 18(1). doi: 10.1186/S12970-020-00383-4. PMC7777221 33388079

[5] Salinero JJ, Lara B, Del Coso J. Effects of acute ingestion of caffeine on team sports performance: a systematic review and meta-analysis. Res Sports Med. 2019; 27(2):238–56. doi: 10.1080/15438627.2018.1552146. 30518253

[6] Baltazar-Martins JG, Brito De Souza D, Aguilar M, Grgic J, Del Coso J. Infographic. The road to the ergogenic effect of caffeine on exercise performance. Br J Sports Med. 2020; 54(10):618–9. doi: 10.1136/BJSPORTS-2019-101018. 31278086

[7] Giraldez-Costas V, Aguilar-Navarro M, Gonzalez-Garcia J, Del Coso J, Salinero JJ. Acute caffeine supplementation enhances several aspects of shot put performance in trained athletes. J Int Soc Sports Nutr. 2022; 19(1):366–80. doi: 10.1080/15502783.2022.2096415. 35813843 PMC9261737

[8] Lara B, Salinero JJ, Giráldez-Costas V, Del Coso J. Similar ergogenic effect of caffeine on anaerobic performance in men and women athletes. Eur J Nutr. 2021; 60(7):4107–14. doi: 10.1007/S00394-021-02510-6. 33606090

[9] Diaz-Lara J, Nieto-Acevedo R, Abian-Vicen J, Del Coso J. Can Caffeine Change the Game? Effects of Acute Caffeine Intake on Specific Performance in Intermittent Sports During Competition: A Systematic Review and Meta-Analysis. Int J Sports Physiol Perform. 2024; 19(11):1180–96. doi: 10.1123/ijspp.2023-0232. 39168455

[10] Grgic J, Grgic I, Pickering C, Schoenfeld BJ, Bishop DJ, Pedisic Z. Wake up and smell the coffee: caffeine supplementation and exercise performance-an umbrella review of 21 published meta-analyses. Br J Sports Med. 2020; 54(11):681–8. doi: 10.1136/bjsports-2018-100278. 30926628

[11] Kaasinen V, Aalto S, Nagren K, Rinne JO. Expectation of caffeine induces dopaminergic responses in humans. Eur J Neurosci. 2004; 19(8):2352–6. doi: 10.1111/j.1460-9568.2004.03310.x. 15090062

[12] Dawkins L, Shahzad F-Z, Ahmed SS, Edmonds CJ. Expectation of having consumed caffeine can improve performance and mood. Appetite. 2011; 57(3):597–600. doi: 10.1016/j.appet.2011.07.011. 21824504

[13] Ortiz-Sánchez D, Bravo-Sánchez A, Ramírez-delaCruz M, Abián P, Abián-Vicén J, Ortiz-Sánchez D, et al. Placebo Effect of Caffeine on Physiological Parameters and Physical Performance. Nutrients. 2024; 16(10):1405. doi: 10.3390/nu16101405. 38794643 PMC11123970

[14] Hurst P, Schipof-Godart L, Hettinga F, Roelands B, Beedie CJ. Improved 1000-m Running Performance and Pacing Strategy With Caffeine and Placebo: A Balanced Placebo Design Study. Int J Sports Physiol Perform. 2019; 15(4):483–8. doi: 10.1123/IJSPP.2019-0230. 31575826

[15] Miller FG, Wendler D, Swartzman LC. Deception in research on the placebo effect. PLoS Med. 2005; 2(9):e262. doi: 10.1371/journal.pmed.0020262. 16173830 PMC1198039

[16] Foad AJ, Beedie CJ, Coleman DA. Pharmacological and psychological effects of caffeine ingestion in 40-km cycling performance. Med Sci Sports Exerc. 2008; 40(1):158–65. doi: 10.1249/mss.0b013e3181593e02. 18091009

[17] Valero F, González-Mohíno F, Salinero JJ. Belief That Caffeine Ingestion Improves Performance in a 6-Minute Time Trial Test without Affecting Pacing Strategy. Nutrients. 2024; 16(2):327. doi: 10.3390/nu16020327. 38276565 PMC10819016

[18] Filip-Stachnik A, Krzysztofik M, Kaszuba M, Leońska-Duniec A, Czarny W, Del Coso J, et al. Placebo Effect of Caffeine on Maximal Strength and Strength Endurance in Healthy Recreationally Trained Women Habituated to Caffeine. Nutrients. 2020; 12(12):3813. doi: 10.3390/nu12123813. 33322129 PMC7763627

[19] Pérez-López A, Salinero JJ, Abian-Vicen J, Valadés D, Lara B, Hernandez C, et al. Caffeinated energy drinks improve volleyball performance in elite female players. Med Sci Sports Exerc. 2015; 47(4):850–6. doi: 10.1249/MSS.0000000000000455. 25051390

[20] McDermott BP, Anderson SA, Armstrong LE, Casa DJ, Cheuvront SN, Cooper L, et al. National Athletic Trainers’ Association Position Statement: Fluid Replacement for the Physically Active. J Athl Train. 2017; 52(9):877–95. doi: 10.4085/1062-6050-52.9.02. 28985128 PMC5634236

[21] Burke LM, Hawley JA, Wong SH, Jeukendrup AE. Carbohydrates for training and competition. J Sports Sci. 2011; 29 Suppl 1:S17–27. doi: 10.1080/02640414.2011.585473. 21660838

[22] Burke LM, Castell LM, Casa DJ, Close GL, Costa RJS, Desbrow B, et al. International Association of Athletics Federations Consensus Statement 2019: Nutrition for Athletics. Int J Sport Nutr Exerc Metab. 2019; 29(2):73–84. doi: 10.1123/ijsnem.2019-0065. 30952204

[23] Bühler E, Lachenmeier DW, Schlegel K, Winkler G. Development of a tool to assess the caffeine intake among teenagers and young adults. Ernähr Umsch. 2014; 61(4):58–63.

[24] Filip A, Wilk M, Krzysztofik M, Del Coso J. Inconsistency in the Ergogenic Effect of Caffeine in Athletes Who Regularly Consume Caffeine: Is It Due to the Disparity in the Criteria That Defines Habitual Caffeine Intake? Nutrients. 2020; 12(4):1087. doi: 10.3390/nu12041087. 32326386 PMC7230656

[25] Multhuaptff W, Fernández-Peña E, Moreno-Villanueva A, Soler-López A, Rico-González M, Manuel Clemente F, et al. Concurrent-Validity and Reliability of Photocells in Sport: A Systematic Review. J Hum Kinet. 2023; 92:53–71. doi: 10.5114/jhk/174285. 38736609 PMC11079923

[26] Koo TK, Li MY. A Guideline of Selecting and Reporting Intraclass Correlation Coefficients for Reliability Research. J Chiropr Med. 2016; 15(2):155–63. doi: 10.1016/j.jcm.2016.02.012. 27330520 PMC4913118

[27] Salinero JJ, Lara B, Abian-Vicen J, Gonzalez-Millán C, Areces F, Gallo-Salazar C, et al. The use of energy drinks in sport: Perceived ergogenicity and side effects in male and female athletes. Br J Nutr. 2014; 112(9):1494–502. doi: 10.1017/S0007114514002189. 25212095

[28] Hopkins WG. A Scale of Magnitudes for Effect Statistics 2016. Available from: http://www.sportsci.org/resource/stats/.

[29] Saunders B, de Oliveira LF, da Silva RP, de Salles Painelli V, Gonçalves LS, Yamaguchi G, et al. Placebo in sports nutrition: a proof-of-principle study involving caffeine supplementation. Scand J Med Sci Sports. 2017; 27(11):1240–7. doi: 10.1111/SMS.12793. 27882605

[30] Costa G, Galvão L, Bottaro M, Mota JF, Pimentel GD, Gentil P. Effects of placebo on bench throw performance of Paralympic weightlifting athletes: a pilot study. J Int Soc Sports Nutr. 2019; 16(1). doi: 10.1186/s12970-019-0276-9. PMC6381705 30782172

[31] Beedie CJ, Stuart EM, Coleman DA, Foad AJ. Placebo effects of caffeine on cycling performance. Med Sci Sports Exerc. 2006; 38(12):2159–64. doi: 10.1249/01.mss.0000233805.56315.a9. 17146324

[32] Anderson DE, German RE, Harrison ME, Bourassa KN, Taylor CE. Real and Perceived Effects of Caffeine on Sprint Cycling in Experienced Cyclists. J Strength Cond Res. 2020; 34(4):929–33. doi: 10.1519/JSC.0000000000003537. 31996613

[33] Del Coso J, Lara B, Ruiz-Moreno C, Salinero JJ. Challenging the myth of non-response to the ergogenic effects of caffeine ingestion on exercise performance. Nutrients. 2019; 11(4). doi: 10.3390/NU11040732. PMC6521624 30934886

[34] Lara B, Ruiz-Vicente D, Areces F, Abián-Vicén J, Salinero JJ, Gonzalez-Millán C, et al. Acute consumption of a caffeinated energy drink enhances aspects of performance in sprint swimmers. Br J Nutr. 2015; 114(6):908–14. doi: 10.1017/S0007114515002573. 26279580

[35] Graham T, Spriet L. Metabolic, catecholamine, and exercise performance responses to various doses of caffeine. J Appl Physiol. 1995; 78(3). doi: 10.1152/jappl.1995.78.3.867. 7775331

[36] Lara B, Ruiz-Moreno C, Salinero JJ, Coso JD. Time course of tolerance to the performance benefits of caffeine. PloS One. 2019; 14(1). doi: 10.1371/JOURNAL.PONE.0210275. PMC6343867 30673725

[37] Ruiz-Moreno C, Lara B, Salinero JJ, Brito de Souza D, Ordovás JM, Del Coso J. Time course of tolerance to adverse effects associated with the ingestion of a moderate dose of caffeine. Eur J Nutr. 2020; 59(7):3293–302. doi: 10.1007/S00394-019-02167-2. 31900579

[38] Lara B, Gutiérrez Hellín J, Ruíz-Moreno C, Romero-Moraleda B, Del Coso J. Acute caffeine intake increases performance in the 15-s Wingate test during the menstrual cycle. Br J Clin Pharmacol. 2020; 86(4):745–52. doi: 10.1111/BCP.14175. 31747465 PMC7098873

[39] Lara B, Gutiérrez-Hellín J, García-Bataller A, Rodríguez-Fernández P, Romero-Moraleda B, Del Coso J. Ergogenic effects of caffeine on peak aerobic cycling power during the menstrual cycle. Eur J Nutr. 2020; 59(6):2525–34. doi: 10.1007/S00394-019-02100-7. 31691019

[40] Romero-Moraleda B, Coso JD, Gutiérrez-Hellín J, Ruiz-Moreno C, Grgic J, Lara B. The Influence of the Menstrual Cycle on Muscle Strength and Power Performance. J Hum Kinet. 2019; 68(1):123–33. doi: 10.2478/HUKIN-2019-0061. 31531138 PMC6724592

